# *Dental Dynamics*: A Fast New Tool for Quantifying Tooth and Jaw Biomechanics in 3D Slicer

**DOI:** 10.1093/iob/obae015

**Published:** 2024-05-10

**Authors:** K E Cohen, A R Fitzpatrick, J M Huie

**Affiliations:** California State University Fullerton, Biological Science, Fullerton, CA 98231, USA; Department of Biology, University of Florida, Gainesville, FL 32611, USA; University of Florida, Museum of Natural History, Gainesville, FL 32611, USA; Department of Biology, University of Florida, Gainesville, FL 32611, USA; Department of Biology, The George Washington University, Washington, DC 20052, USA

## Abstract

Teeth reveal how organisms interact with their environment. Biologists have long looked at the diverse form and function of teeth to study the evolution of feeding, fighting, and development. The exponential rise in the quantity and accessibility of computed tomography (CT) data has enabled morphologists to study teeth at finer resolutions and larger macroevolutionary scales. Measuring tooth function is no easy task, in fact, much of our mechanical understanding is derived from dental shape. Categorical descriptors of tooth shape such as morphological homodonty and heterodonty, overlook nuances in function by reducing tooth diversity for comparative analysis. The functional homodonty method quantitatively assesses the functional diversity of whole dentitions from tooth shape. This method uses tooth surface area and position to calculate the transmission of stress and estimates a threshold for functionally homodont teeth through bootstrapping and clustering techniques. However, some vertebrates have hundreds or thousands of teeth and measuring the shape and function of every individual tooth can be a painstaking task. Here, we present *Dental Dynamics*, a module for 3D Slicer that allows for the fast and precise quantification of dentitions and jaws. The tool automates the calculation of several tooth traits classically used to describe form and function (i.e., aspect ratio, mechanical advantage, force, etc.). To demonstrate the usefulness of our module we used *Dental Dynamics* to quantify 780 teeth across 20 salamanders that exhibit diverse ecologies. We coupled these data with the functional homodonty method to investigate the hypothesis that arboreal *Aneides* salamanders have novel tooth functions. *Dental Dynamics* provides a new and fast way to measure teeth and increases the accessibility of the functional homodonty method. We hope *Dental Dynamics* will encourage further theoretical and methodological development for quantifying and studying teeth.

## Abstract (Spanish)

Los dientes revelan cómo los organismos interactúan con su entorno. Los biólogos llevan mucho tiempo observando las diversas formas y funciones de los dientes para estudiar la evolución de la alimentación, la lucha y el desarrollo. El aumento exponencial en la cantidad y accesibilidad de los datos de la tomografía computarizada (CT) ha permitido a los morfólogos estudiar los dientes con resoluciones más finas y escalas macroevolutivas más grandes. Medir la función dental no es una tarea fácil; de hecho, gran parte de nuestro conocimiento mecánico se deriva de la forma dental. Los descriptores categóricos de la forma de los dientes, como la homodoncia y la heterodoncia morfológicas, pasan por alto los matices funcionales al reducir la diversidad de los dientes para el análisis comparativo. El método de la homodoncia funcional cuantitativamente evalúa la diversidad funcional de denticiones enteras a partir de la forma del diente. Sin embargo, algunos vertebrados tienen cientos o miles de dientes y medir la forma y función de cada uno de los dientes individuales puede ser una tarea minuciosa. Aquí te presentamos Dental Dynamics, un módulo para 3D Slicer que permite la cuantificación rápida y precisa de denticíon y mandíbulas. La herramienta automatiza el cálculo de varios rasgos dentales utilizados clásicamente para describir forma y función (es decir, relación de aspecto, ventaja mecánica, fuerza, etc.). Para demostrar la utilidad de nuestros módulos utilizamos Dental Dynamics para cuantificar 780 dientes en 20 salamandras que exhiben ecologías diversas. Combinamos estos datos con el método de homodoncia funcional para investigar la hipótesis de que las salamandras arbóreas Aneides tienen nuevas funciones dentales. Dinámica Dental proporciona una forma nueva y rápida de medir los dientes y aumenta la accesibilidad de los método funcionales de homodoncia. Esperamos que Dental Dynamics fomente más conocimientos teóricos y desarrollo metodológico para la cuantificación y estudio de los dientes.

## Abstract (German)

Zähne enthüllen, wie Organismen mit ihrer Umwelt interagieren. Biolog: innen untersuchen seit langem die diverse Form und Funktion von Zähnen, um die Evolution von Nahrungsaufnahme, Kampf und Entwicklung zu entschlüsseln. Der exponentielle Anstieg in der Menge und dem Zugang zu Computertomografiedaten (CT-Daten) hat es Morpholog: innen ermöglicht, Zähne mit höherer Auflösung und auf größeren makroevolutionären Skalen zu erforschen. Zahnfunktion zu messen ist keine leichte Aufgabe, viele unserer mechanischen Erkenntnisse haben wir von der Zahnform. Kategorische

Beschreibung von Zahnform wie morphologische Homo- und Heterodontie übersehen funktionelle Nuancen, indem sie Zahndiversität für vergleichende Analysen reduzieren. Die Methode der funktionellen Homodontie erfasst quantitativ die funktionelle Diversität ganzer Gebisse mittels der Zahnform. Diese Methode verwendet Zahnoberfläche und Position, um die Leitung von Stress zu berechnen und schätzt einen Schwellenwert für funktionell homodonte Zähne durch Bootstrapping und Clustering-Techniken ab. Allerdings haben einige Vertebraten hunderte oder tausende Zähne und das Messen von Form und Funktion jedes einzelnen Zahns kann mühsam sein. Wir präsentieren hier *Dental Dynamics*, ein Modul für 3D Slicer, welches eine schnelle und präzise Quantifizierung von Gebissen und Kiefern ermöglicht. Das Modul automatisiert die Berechnung von mehreren klassischerweise für die Beschreibung von Zahnform und -funktion verwendeten Merkmalen (z.B. aspect ratio, mechanical advantage, force etc.). Um den Nutzen unseres Moduls zu demonstrieren, haben wir *Dental Dynamics* verwendet, um 780 Zähne von 20 Salamandern mit diverser Ökologie zu quantifizieren. Wir haben diese Daten mit der funktionellen Homodontie gepaart, um die Hypothese zu erforschen, dass arboreale *Aneides-*Salamander neuartige Zahnfunktion haben. *Dental Dynamics* bietet eine neue, schnelle Art, Zähne zu messen und erhöht die Zugänglichkeit der funktionellen Homodontie-Methode. Wir hoffen, dass *Dental Dynamics* weitere theoretische und methodologische Entwicklungen für die Quantifikation und das Studium von Zähnen inspiriert.

## Abstract (he)

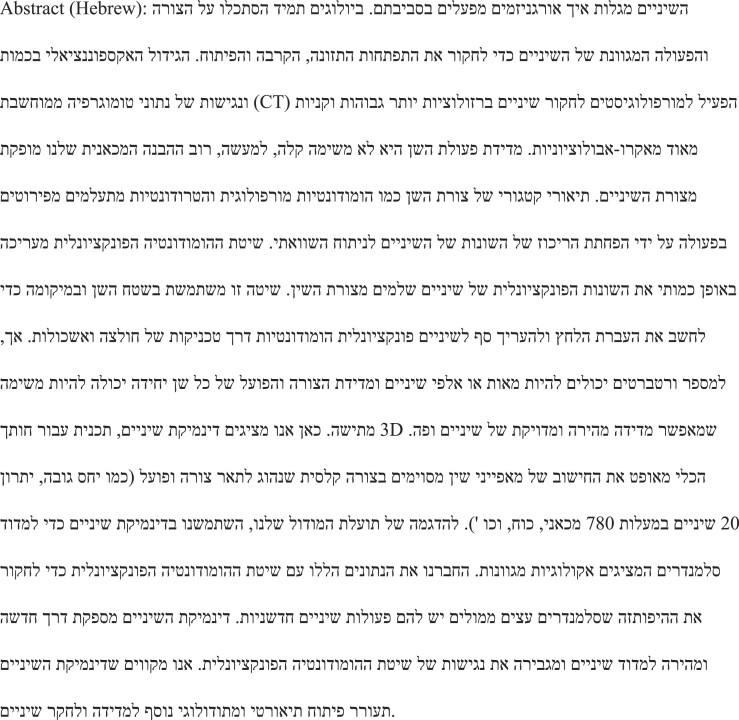

## Introduction

Show me your teeth and I will tell you who you are (Cuvier et al. 1812; Rudwick 2008). Anatomists have long been enamored by our ability to identify species, infer tooth function, and diet from dental shapes and organization of dentitions (Summers 2000; Whitenack 2008; Ungar 2010; Crofts and Summers 2014; Conway et al. 2015; D'Amore 2015; Corn et al. 2016; Huie et al. 2019; Kolmann et al. 2019, 2021; Cohen, Weller, and Summers 2020; Heiple et al. 2023; Segall et al. 2023). The enamel composition of teeth makes them predisposed to fossilize, creating a record of their shape and function across space and time (Dean 1987, 2000). In fact, the seafloor is littered with teeth and odontodes of extant and extinct species allowing us to reconstruct the composition of food webs and ecosystems that existed millions of years ago (Sibert et al. 2014; Sibert and Norris 2015). The deciduous teeth of many vertebrates bear the lasting scars of function as their wear and tear reveal damage (Ungar and Williamson 2000; Carr et al. 2006; Christiansen et al. 2010; Ungar et al. 2010; Collins and Underwood 2021; Huysseune and Witten 2023), and the organizational structure of hydroxyapatite crystals reflect mechanical stress during prey loading (Enax et al. 2014; Marcus et al. 2017; Werth et al. 2019; Delaunois et al. 2020; Deng et al. 2022). Teeth from today, yesterday, or 400 million years ago provide answers to the questions of what animals eat and how they interact with each other. And while tooth morphology has resulted in endless interpretations of diet, phylogeny, and usage; quantifying tooth function remains a challenge.

Shape-based predictions of tooth function alone offer limited insights into the diversity of dentitions (D'Amore 2015; Mihalitsis and Bellwood 2019; Cohen, Weller, and Summers 2020, Cohen, Weller, Westneat et al. 2020; Huie et al. 2020; Hulsey et al. 2020; Ryerson and Van Valkenburg 2021). Homodonty is a classic anatomical descriptor of tooth shape where all teeth in the dental battery are of the same shape or size, such as a series of conical teeth in most bony fish (Simpson 1936; Keene 1991; Ungar 2010). Heterodonty is reserved for clearly regionalized morphology, which describes a typical mammalian jaw with incisors, canines, premolars, and molars which have been frequently shown to have distinct biomechanical capabilities. Molars are well known for their ability to crush and fracture prey, while canines pierce through flesh. Now, imagine a dental battery lined with only canines—do the posterior canines still puncture or do they take on a new crushing behavior? There are no strict rules for tooth shape and function; rather, they exist on a continuum. However, qualitative classifications like morphological homodont, macrodont, or edentulate fail to encompass the full range of functional variation by simplifying dentitions for comparative analyses (Mihalitsis and Bellwood 2019; Cohen, Weller, and Summers 2020, Cohen, Weller, Westneat et al. 2020). It is easy to overlook the functional diversity hidden in a battery of conical teeth. Under a morphological homodonty classification, the many conical teeth on a piscivorous lingcod jaw are all perceived to be for stabbing. In reality, these fish have coordinating patches of large and small teeth generate functional regionalization comparable to mammals (Cohen, Weller, and Summers 2020; Carr et al. 2021). We have many powerful tools for quantifying tooth shapes (Robinson et al. 2002; Anderson and LaBarbera 2008; Whitenack and Motta 2010; Bunn et al. 2011; Santana et al. 2011; Pampush et al. 2016; Anderson et al. 2019; Crofts et al. 2020), however, to directly measure dental function, we need to look beyond the morphology of isolated teeth and instead consider the biomechanics of the entire jaw.

The functional homodonty method quantitatively assesses the functional diversity of dentitions by calculating the stress each tooth can transmit to prey based on its surface area and position along the jaw (Cohen, Weller, and Summers 2020, Cohen, Weller, Westneat et al. 2020). Variation in stresses reveal a functional homodont-heterodont continuum that can be studied across species and analyzed in an evolutionary framework. The functional homodonty method enables us to describe the functional diversity of whole dentitions, how tooth clusters work in tandem, and isolate functionally unique teeth on a jaw. For example, *Halichoeres* wrasses are abundant reef fish with dentitions of small conical teeth in very similar arrangements (Cohen, Weller, Westneat, et al. 2020). From a morphological perspective, there should be little functional variation in how these teeth interact with prey and yet, functional heterodonty has evolved at least three times. Some fish have dentitions like the lingcod where large and small teeth work together in transmitting forces. Others have one particular tooth—a large central canine—that exerts stresses 12 times greater than any other tooth on the jaw. Each instance of functional heterodonty reveals nuances in how this group of wrasses interact with their prey (Cohen, Weller, Westneat et al. 2020).

One of the major barriers to studying teeth is resolution. Large animals are convenient because they have large teeth, but smaller animals are more difficult to study with conventional imaging techniques. Micro-computed tomography (micro-CT) scanning has become a popular and highly effective method for conducting morphological investigations on dental batteries (Kolmann et al. 2018, 2019; Buser et al. 2020; Williams et al. 2022; Segall et al. 2023). It produces high-resolution, three-dimensional data that can be digitally dissected without damaging the physical specimens. The advantages extend to fossils that may be embedded in a matrix and would otherwise be difficult to examine (Wu and Schepartz 2009; Abel et al. 2012; O'Hara et al. 2019). Furthermore, the availability of computed tomography (CT) data; both in terms of taxonomic diversity and accessibility, has grown exponentially in the last two decades. Initiatives like MorphoSource, The Open Vertebrate Project (OVERT), or #ScanAllFishes provide access to thousands of vertebrate scans (Boyer et al. 2016; Davies et al. 2017; Rolfe et al. 2021). However, accessibility is a double-edged sword. Some vertebrates have hundreds of teeth, and our ability to study whole dental batteries on macroevolutionary scales is hindered by processing time and technology. Thus, we are in dire need of new software that can aid in the rapid collection of morphological data from CT scans.

A promising platform for rapid CT data quantification is the open-source image computing software 3D Slicer, which has been designed to visualize and analyze 2D, 3D, and 4D data (Kikinis et al. 2014; Rolfe et al. 2021). It is available across operating systems, and comes equipped with a powerful suite of tools for manual and semi-automatic segmentation, measuring and manipulating 3D data, and data visualization. Functions that are not provided by the core environment can be developed by external users and uploaded to the application's native extension manager. Recently, 3D Slicer has gained a lot of traction among organismal biologists and morphologists due to the development of recent tutorials, tools, and extensions (*SlicerMorph, SegmentGeometry, MEMOS*, etc.) (Buser et al. 2020; Rolfe et al. 2021; Huie et al. 2022; Rolfe et al. 2023). These have helped make 3D Slicer a one-stop-shop for processing and reusing CT data.

**Fig. 1:**
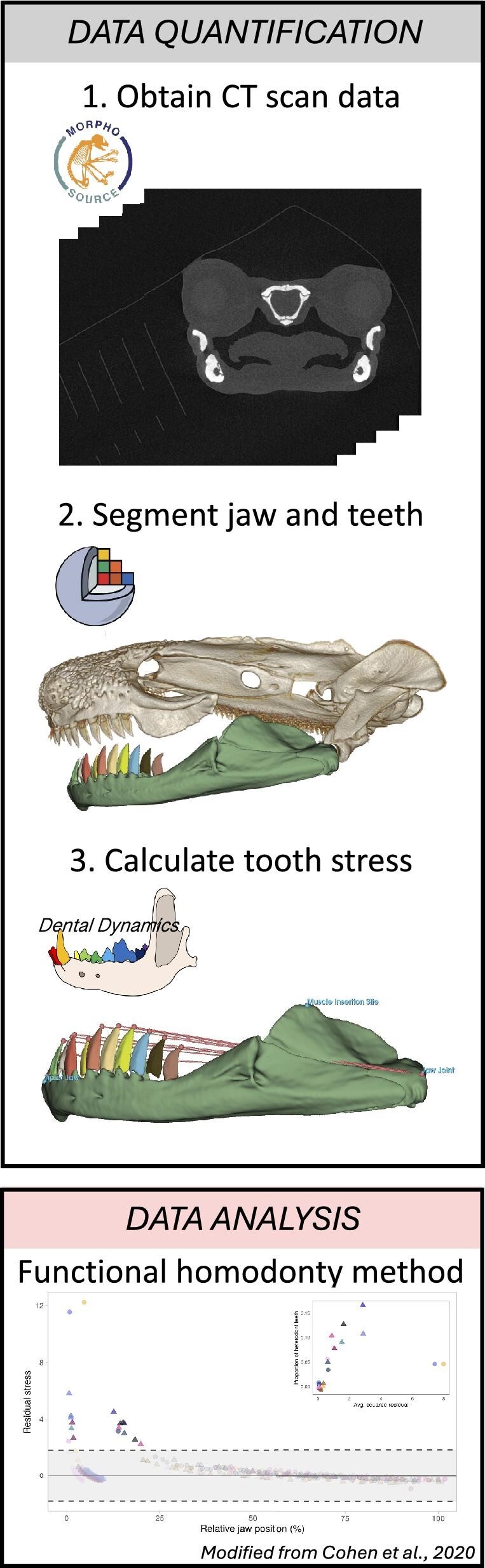
Workflow for the quantification of tooth and jaw biomechanics in 3D Slicer. (1) Obtain CT scan data from an open-source repository. (2) Segment the jaws and teeth in 3D Slicer using native segmentation tools. (3) Use the new 3D slicer module *Dental Dynamics* to calculate the jaw and tooth traits (i.e., aspect ratio, output force, tooth stress, etc.). One of the ways to analyze results from *Dental Dynamics* is to use the functional homodonty method (Cohen, Weller, and Summers 2020; Cohen, Weller, Westneat et al.  2020) to assess the functional variation of whole dentitions.

Here, we present *Dental Dynamics*, a new module for 3D Slicer designed to model tooth function in vertebrate jaws (Fig. 1). Below, we describe the utility of *Dental Dynamics* and how it automates the calculation of several key jaw and tooth traits that can be directly exported for the calculation of functional homodonty and other analyses of jaw biomechanics. To demonstrate the efficacy of *Dental Dynamics*, we present a use case on the evolution of tooth function in plethodontid salamanders. Our module is capable of quantifying dozens of teeth in seconds allowing for the efficient collection of highly valuable morphological data.

## Dental Dynamics for 3D Slicer

*Dental Dynamics* is a Python-based module for 3D Slicer designed to run on the current stable release of the program (version 5.6.1, r32438). The module is included in the new *SlicerBiomech* extension, which also contains the pre-existing *SegmentGeometry* module (formerly in the *SegmentGeoemtry* extension) for calculating second moment of area and other cross-sectional traits (Huie et al. 2022). The official method of obtaining *Dental Dynamics* is by downloading and installing the *SlicerBiomech* extension through Slicer's built-in extension manager. Detailed documentation about *Dental Dynamics*, step-by-step instructions on how to install and use *Dental Dynamics*, and the source code are all provided on a GitHub repository (https://github.com/jmhuie/SlicerBiomech).

### Functionality

**Fig. 2:**
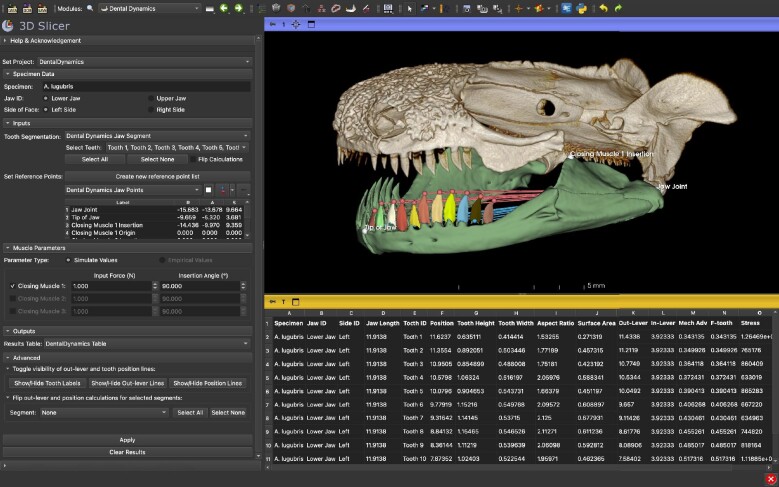
The *Dental Dynamics* graphical user interface in 3D Slicer. The module enables the user to enter specimen metadata, define anatomical landmarks on the jaw, edit modeling parameters, and calculate several jaw and tooth traits. Shown is the skull of *Aneides lugubris* with the lower jaw and individual teeth segmented out. The red lines (upper set of lines) from the jaw joint to the tips of the teeth represent the out levers used to calculate mechanical advantage. The length of the blue lines (lower set of lines) connecting the jaw joint to the bases of the teeth represent the position of each tooth along the jaw. An example results table is shown, which can be saved and imported into statistical analysis software.

*Dental Dynamics* is designed to calculate several jaw and tooth traits using a 3D Slicer segmentation file containing individually segmented teeth and user-defined anatomical landmarks (jaw joint, tip of the jaw, and the insertion and origin site of up to three jaw closing muscles). These inputs are used to automatically calculate jaw length and muscle in-levers, and for each tooth its position along the jaw, height, width, aspect ratio, surface area, mechanical advantage, output force, and tooth stress. *Dental Dynamics* can also take additional user inputs to estimate muscle parameters such as input force and insertion angle to provide more informed estimates of bite force. The module is equipped with a graphical user interface that enables the editing of specimen metadata, selecting the necessary input files and points, and adjusting the additional muscle parameters. Once results have been computed, they are presented in a table that can be readily imported into other statistical software (Fig. 2). Although the intended use cases involve computing on CT segmentations made in 3D Slicer, segmentations and meshes (.obj, .ply, .stl) produced by other software can be easily converted into a usable format with native 3D Slicer functionalities.

The stress that a tooth can transmit to prey is calculated by taking the force a tooth can exert and dividing it by the surface area of the tooth


\begin{eqnarray*}
\sigma = \frac{{{{F}_{\mathit{tooth}}}}}{{S{{A}_{\mathit{tooth}}}}}.\end{eqnarray*}

Assuming the tooth resembles a cone, the surface area of the tooth can be approximated using the height and radius of the tooth. However, teeth are rarely perfect cones. They are often curved, asymmetrical, or deviate from the conical shape altogether. *Dental Dynamic*s calculates the surface area of a tooth with any geometric shape more accurately by using the native 3D Slicer surface area calculator in the *Segment Statistics* module (Kikinis et al. 2014). Because tooth aspect ratio can still provide relevant information separate from surface area and stress, *Dental Dynamic*s will still measure the height and width of each tooth.

Jaws are often modeled as simple lever systems (Westneat 2003, 2004) and the forces that teeth can exert depend on their position along the jaw. The force exerted by a given tooth on the jaw can be calculated as


**Fig. 3:**
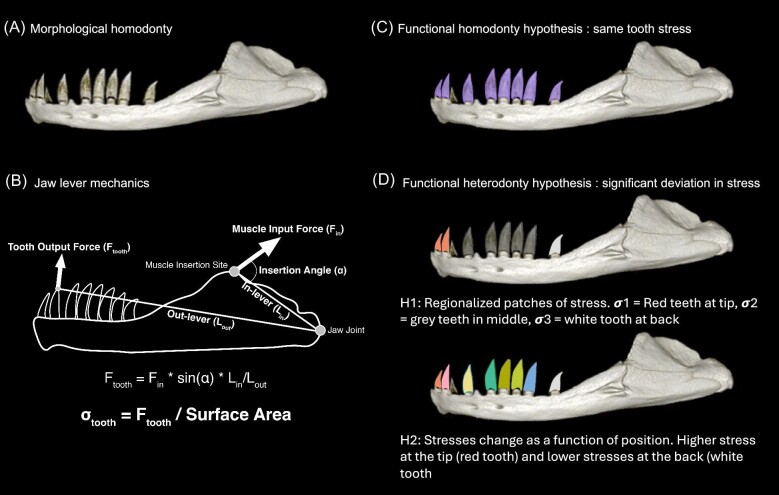
Functional vs. morphological homodonty. (A) Morphological homodonty represents a morphology where all teeth are the same shape or size along the jaw. (B) Tooth function is modeled using simple lever mechanics to estimate how much force and stress a tooth can exert based on its surface area and position on the jaw. (C) Functional homodonty is represented by all teeth bearing the same stress. All teeth are colored purple indicating they may have similar stress values. (D) Functional heterodonty exists on a continuum and represents significant variations in stress along the jaw. Two functional heterodonty hypotheses are represented here. The first condition demonstrates functional regionalization. All gray teeth have similar stress values that differ from the red teeth at the tip and white teeth at the back. The second condition represents a gradient of stresses that match our prediction based on jaw lever mechanics with teeth at the front bearing lesser stress values than those at the back.

\begin{eqnarray*}
{{F}_{\mathit{tooth}}} = {{F}_{in}}*{\rm sin}\left( \alpha \right)*\left( {\frac{{in\ \mathit{lever}}}{{out\ \mathit{lever}}}} \right),\end{eqnarray*}

where *F_in_* is the input force of a jaw closing muscle, ɑ is the angle of insertion for that muscle in degrees, in lever is the distance from the jaw joint to the muscle insertion site, and out lever is the distance between the jaw joint and the tip of the tooth (Fig. 3). By default, *Dental Dynamics* will assume a static bite force of 1 N and muscle insertion angle of 90°, causing tooth force estimates to reflect variation in mechanical advantage (in lever/out lever). However, the input force and angle of insertion of each closing muscle can be manipulated with the user interface to perform simulations under different conditions. When multiple closing muscles are used to calculate output force, the *F_tooth_* calculated for each muscle are summed together to achieve a total force used to calculate tooth stress.

If the anatomy of the jaw closing muscles are known, either through dissection or CT scans with contrast stain, users can calculate more biologically informed muscle parameters and output values. The user interface allows for the landmarking of the origin of each closing muscle that can be used to help measure the angle of insertion. Muscle input force can be estimated by calculating the physiological cross-sectional area and multiplying it by maximal isometric stress (*F*_max_) (Martin et al. 2020). Using user-provided estimates of muscle volume, input force can be calculated as


\begin{eqnarray*}
{{F}_{in}}\ = \ \frac{{\mathit{volume}*{\rm cos}\left( \alpha \right)}}{{\mathit{fiber}\ \mathit{length}}}*\ {{F}_{\rm max}},\end{eqnarray*}

where α is the pennation angle of the muscle fibers in degrees. *Dental Dynamics* uses the distance between a muscle's origin and insertion as a proxy for fiber length, and by defaults, assumes a pennation angle of 0°. The maximal isometric stress for vertebrate muscles can vary based on taxon and region of the body (Westneat 2003; Martin et al. 2020). However, *Dental Dynamics* assumes a default *F*_max_ value of 0.2 N/mm^2^ to calculate input force, like MandibLever (Westneat 2003). Both the pennation angle and the *F*_max_ values can be modified and defined by the user. *Dental Dynamics* uses the provided jaw metadata and anatomical landmarks to automatically determine where the tip and base of each tooth is. First, *Dental Dynamics* identifies the principal axis that runs along the length of a tooth and crosses the centroid. Two landmarks are placed on the principal axis at either ends of the tooth. The landmark closer to a given point along a vector defined by the jaw tip and jaw joint is assumed to be closer to the base of the tooth. To find the center of the base, the base landmark is slid along the principal axis until it contacts the tooth. The tip landmark is raised above the tooth and then snapped to the nearest point on the surface, which should be the tip of the tooth. The positions of these landmarks are determined for each tooth, used in the calculations, and saved into their own point lists. After initially computing results, users can manually adjust the position of the points and recompute the results. In instances where *Dental Dynamics* may incorrectly identify the tip and base of some or all teeth, the user interface is equipped with tools to flip the landmark positions for all teeth or just selected ones. Additional quality control tools are provided such as the ability to toggle on and off the display of tooth labels and lines connecting the jaw joint to the tooth tips and bases.

### Limitations

*Dental Dynamics* is a useful tool for automating many calculations performed on teeth but has some caveats that are worth considering. Our module is optimized for calculating stress from teeth with a singular point (i.e., cones). For the module to work on multi-cuspid dentition, a single consensus point must be chosen to calculate mechanical advantage and tooth stress. That reflects a limitation in our models for estimating tooth stress and jaw leverage, but with more user input a solution on handling multi-cuspid dentition may be devised. Currently, *Dental Dynamics* is limited to modeling static bites forces and does not take factors such as jaw angle and speed of jaw closure into account. The module also assumes that there are no lateral or antero-posterior displacements during a bite, a common phenomenon during mastication. Further development of the module could consist of providing support for modeling tooth stress under more dynamic conditions. Lastly, *Dental Dynamics* requires the teeth to be pre-segmented, a time-consuming process that exists independently from the module. The recent and rapid development of AI and auto-segmentation tools will likely provide a solution in the near future.

## Case study: Evolution of heterodonty in plethodontid salamanders

### Tooth function of plethodontid salamanders

Habitat influences an animal's diet and feeding strategy by limiting prey availability or imposing physical constraints (Diehl 1993; Collar and Wainwright 2009). Thus, habitat transitions may be accompanied by changes in tooth morphology. Salamanders occupy a wide range of environments that span the aquatic-terrestrial gradient, and a number of terrestrial microhabitats (i.e., fossorial, arboreal, and saxicolous). Salamanders also exhibit a range of diverse tooth shapes, numbers, and positions (Caldwell and Trauth 1979; Beneski and Larsen 1989; Gregory et al. 2016). To capture prey, aquatic salamanders typically rely on suction feeding, but on land many species use a combination of biting and, to varying extents, high-power tongue projection to pull prey into their mouths (Deban and Wake 2000; Deban et al. 2020). Salamander tooth shape is somewhat correlated with dietary specialization (Gregory et al. 2016), but *how* they feed may be an equally important determinant of shape.

Lungless salamanders (Plethodontidae) have repeatedly invaded arboreal niches (McEntire 2016; Baken and Adams 2019). Most arboreal salamanders have radiated in the tropics, but the temperate genus of *Aneides* salamanders has successfully invaded the arboreal niche, with one species found as high as 88 m above the ground (Spickler et al. 2006; McEntire 2016). *Aneides* salamanders have been characterized as having highly modified dentitions, with fewer and larger teeth compared with other closely-related species (Wake 1963). However, these morphological changes do not appear to be associated with changes in diet. Instead, it has been proposed that the modified dentitions may yield functional adaptations that aid arboreal *Aneides* in securing and manipulating prey more efficiently (Wake 1963; Larson et al. 1981). When feeding, many terrestrial salamanders will swing their heads back and forth after securing and manipulating their prey (Lindquist and Bachmann 1980; Lukanov et al. 2016; pers. obs.). However, performing such violent behaviors on an elevated surface increases the risk of falling. Thus, it may be that the enlarged teeth help *Aneides* to grasp and bite through the tough exoskeleton of arthropods without the need for head shaking (Bury and Martin 1973; Lynch 1985).

The diversity of salamander teeth has not been studied in a quantitative framework. Here, we employ *Dental Dynamics* and the functional homodonty method to assess the functional diversity of teeth in plethodontid jaws. We set out to evaluate whether the morphological changes in *Aneides* teeth are accompanied by functional shifts, and whether those shifts also occur in other arboreal salamander lineages.

## Methods

We downloaded micro-CT data for 20 species from 8 genera of plethodontid salamanders from MorphoSource (https://www.morphosource.org/) that vary in their microhabitats (Table 1). We segmented the lower jaws of each individual in 3D Slicer, and deployed the scissor and island tools to quickly isolate the teeth and separate them into individual segments. We then used *Dental Dynamics* to estimate tooth stresses. Following the provided protocol on GitHub (https://github.com/redacted), we entered the specimen metadata and defined three anatomical landmarks on the jaw. Salamander adductor muscles vary in their relative size and the exact location and area of their insertion sites (Hinderstein 1971; Carroll and Holmes 1980). We did not have adequate information about the muscle anatomy for our sampled salamander species, thus we modeled a simple bite force with a single insertion point on the most dorsal point of the coronoid process, an easily identifiable landmark across species in our study. Jaw and tooth traits were calculated from the left and right sides of the jaw separately for each species. Replacement and highly damaged teeth were ignored, and traits were not calculated.

**Table 1:** List of plethodontid salamander specimens sampled in this study, with their accession numbers and CT metadata.

Species	Museum	Number	Scanning facility	Scan machine	Morphosource Link	Exposure (ms)	Voltage (kV)	Amperage (uA)	Resolution (um)
*Aneides aeneus*	USNM	201021	Smithsonian Institution Bio-Imaging Research (SIBIR) Center	Siemens Somatom Emotion 6-slice scanner	https://www.morphosource.org/media/000085462	200	90	170	14
*Aneides flavipunctatus*	MVZ	123824	UF Nanoscale Research Facility	General Electric phoenix v|tome|x m 240	https://www.morphosource.org/media/000165897	200	70	240	38.2
*Aneides hardii*	MVZ	220872	UF Nanoscale Research Facility	General Electric phoenix v|tome|x m 240	https://www.morphosource.org/media/000165936	200	80	240	28.9
*Aneides lugubris*	USNM	279384	Smithsonian Institution Bio-Imaging Research (SIBIR) Center	Siemens Somatom Emotion 6-slice scanner	https://www.morphosource.org/media/000085413	131	100	170	26.5
*Aneides vagrans*	MVZ	242271	UF Nanoscale Research Facility	General Electric phoenix v|tome|x m 240	https://www.morphosource.org/media/000165925	200	80	240	30.5
*Bolitoglossa lincolni*	UF	172925	UF Nanoscale Research Facility	General Electric phoenix v|tome|x m 240	https://www.morphosource.org/media/000473903	200	60	160	9.3
*Bolitoglossa mexicana*	USNM	573706	Smithsonian Institution Bio-Imaging Research (SIBIR) Center	Siemens Somatom Emotion 6-slice scanner	https://www.morphosource.org/media/000085807	500	100	170	14
*Chiropterotriton chiropterus*	USNM	316224	The University of Texas High-Resolution X-ray Computed Tomography Facility	North Star Imaging NSI Custom build	https://www.morphosource.org/media/000104354	-	120	170	9.6
*Chiropterotriton multidentatus*	USNM	249003	Smithsonian Institution Bio-Imaging Research (SIBIR) Center	Siemens Somatom Emotion 6-slice scanner	https://www.morphosource.org/media/000085999	200	120	140	14
*Chiropterotriton priscus*	USNM	244718	Smithsonian Institution Bio-Imaging Research (SIBIR) Center	Siemens Somatom Emotion 6-slice scanner	https://www.morphosource.org/media/000085997	200	90	170	15
*Desmognathus aureatus*	USNM	155646	Smithsonian Institution Bio-Imaging Research (SIBIR) Center	Siemens Somatom Emotion 6-slice scanner	https://www.morphosource.org/media/000086157	250	80	170	18
*Desmognathus monticola*	AMNH	A-195565	Karel F. Liem Bioimaging Facility of Friday Harbor Labs	Bruker SkyScan 1173	https://www.morphosource.org/media/000561226	500	80	100	18.8
*Desmognathus wrighti*	MHNH	2021.128	Karel F. Liem Bioimaging Facility of Friday Harbor Labs	Bruker SkyScan 1173	https://www.morphosource.org/media/000561221	500	80	100	8.2
*Eurycea multiplicata*	USNM	481114	The University of Texas High-Resolution X-ray Computed Tomography Facility	North Star Imaging NSI Custom build	https://www.morphosource.org/media/000085996	-	120	170	9.6
*Hydromantes samweli*	MVZ	170637	UF Nanoscale Research Facility	General Electric phoenix v|tome|x m 240	https://www.morphosource.org/media/000074072	200	80	200	18.7
*Plethodon caddoensis*	UF	129845	UF Nanoscale Research Facility	General Electric phoenix v|tome|x m 240	https://www.morphosource.org/media/000461188	200	80	180	13.6
*Plethodon punctatus*	UF	174366	UF Nanoscale Research Facility	General Electric phoenix v|tome|x m 240	https://www.morphosource.org/media/000461170	200	80	180	13.6
*Plethodon vehiculum*	UWBM	693	Karel F. Liem Bioimaging Facility of Friday Harbor Labs	Bruker SkyScan 1173	https://www.morphosource.org/media/000561214	500	80	100	12.4
*Thorius minutissimus*	IBH	23011	The University of Texas High-Resolution X-ray Computed Tomography Facility	Zeiss Xradia microXCT-400	https://www.morphosource.org/media/000115607	1000	70	-	4.86
*Thorius tlaxiacus*	MVZ	183447	The University of Texas High-Resolution X-ray Computed Tomography Facility	Zeiss Xradia microXCT-400	https://www.morphosource.org/media/000115617	6000	70	-	4.75

Links to these data on MorphoSource.org are provided.

The outputs from *Dental Dynamics* were exported and collated into a single spreadsheet and analyzed with the functional homodonty method using publicly available R code (https://github.com/hiweller/homodonty_code) adapted to our dataset. The functional homodonty method is described in detail in Cohen *et al*. (2020, 2020), but is outlined briefly here. First, tooth stresses were normalized by the median stress of each jaw to calculate residuals. To determine a threshold for significant variations in residual stress, a bootstrapping analysis was performed per Cohen, Weller, and  Summers (2020). In short, half of the teeth from a dentition were randomly subsampled without replacement and normalized by the subsampled median stress. This procedure was repeated 10,000 times for each dentition to generate a null distribution of residual stress values. A k-medoids clustering analysis (with *n* = 2 clusters) was performed on a random subsample of 5000 residuals 100 times, defining the threshold as the mean of the two resulting cluster centers. Teeth that have residual stresses within the threshold were considered functional homodonts, while those outside of the threshold were functional heterodonts. Dentitions were considered functionally homodont if all their teeth fall within that threshold and exert similar (ideally the same) stress values.

We then generated two metrics from the residual stress values that emphasize specific aspects of the dentition (Cohen, Weller, Westneat et al. 2020). The average squared residual stress captures the degree of functional divergence among teeth from the same battery. Meanwhile, the proportion of functionally heterodont teeth provides insight on whether a battery contains a few unique teeth or several that may imply regionalization. We visualized these continuous traits on a pruned time-calibrated phylogeny (Jetz and Pyron 2018) using the “contMap” function from the phytools package in R (Revell 2012).

## Results

### Performance of Dental Dynamics

We used *Dental Dynamics* to calculate traits for 780 teeth distributed across the left and right lower jaws of 20 different specimens. Computation times were strongly correlated with the number of teeth on each side of the jaw. The time to compute all metrics for a single tooth averaged roughly 0.45 s. On average, the salamanders had roughly 20 teeth on each side of the jaw and *Dental Dynamics* had an average output speed of 8.16 s per side. The number of teeth on a single side of a jaw ranged from 4 to 44, and computing times ranged between 1.45 and 22.18 s, respectively (Table 2). Computation times were recorded and averaged using two computers: a Dell XPS 8950 equipped with a 16-core 12th Gen Intel i9-12900 2.4 GHz processor, a NVIDIA GeForce RTX 3070 GPU, and 64 GB of memory; and a 16-inch 2021 MacBook Pro equipped with an Apple M1 Max chip that has a 10-core CPU, an integrated 32-core GPU, and 64 GB of memory. Computation times were very similar between the two computers, but computations times are expected to vary across machines based on the speed on the processor because computations are performed using the CPU.

**Table 2:** Average tooth counts and computation time data for the 3D Slicer module *Dental Dynamics*.

Species	Average number of teeth	Average computation time (s)
*Aneides aeneus*	6	2.6
*Aneides flavipunctatus*	4	1.8
*Aneides hardii*	8.5	3.2
*Aneides lugubris*	10.5	3.8
*Aneides vagrans*	7	2.9
*Bolitoglossa lincolni*	21.5	12.8
*Bolitoglossa mexicana*	23.5	9.8
*Chiropterotriton chiropterus*	10.5	4.9
*Chiropterotriton multidentatus*	25.5	11.3
*Chiropterotriton priscus*	18.5	8.1
*Desmognathus aureatus*	43	20.1
*Desmognathus monticola*	23.5	8.8
*Desmognathus wrighti*	14.5	6.3
*Eurycea multiplicata*	18	7.9
*Hydromantes samweli*	39	17.1
*Plethodon caddoensis*	39	17.7
*Plethodon punctatus*	33	13.3
*Plethodon vehiculum*	19	7.9
*Thorius minutissimus*	14	5.8
*Thorius tlaxiacus*	11.5	5.3

### Functional heterodonty in plethodontid salamanders

**Fig. 4:**
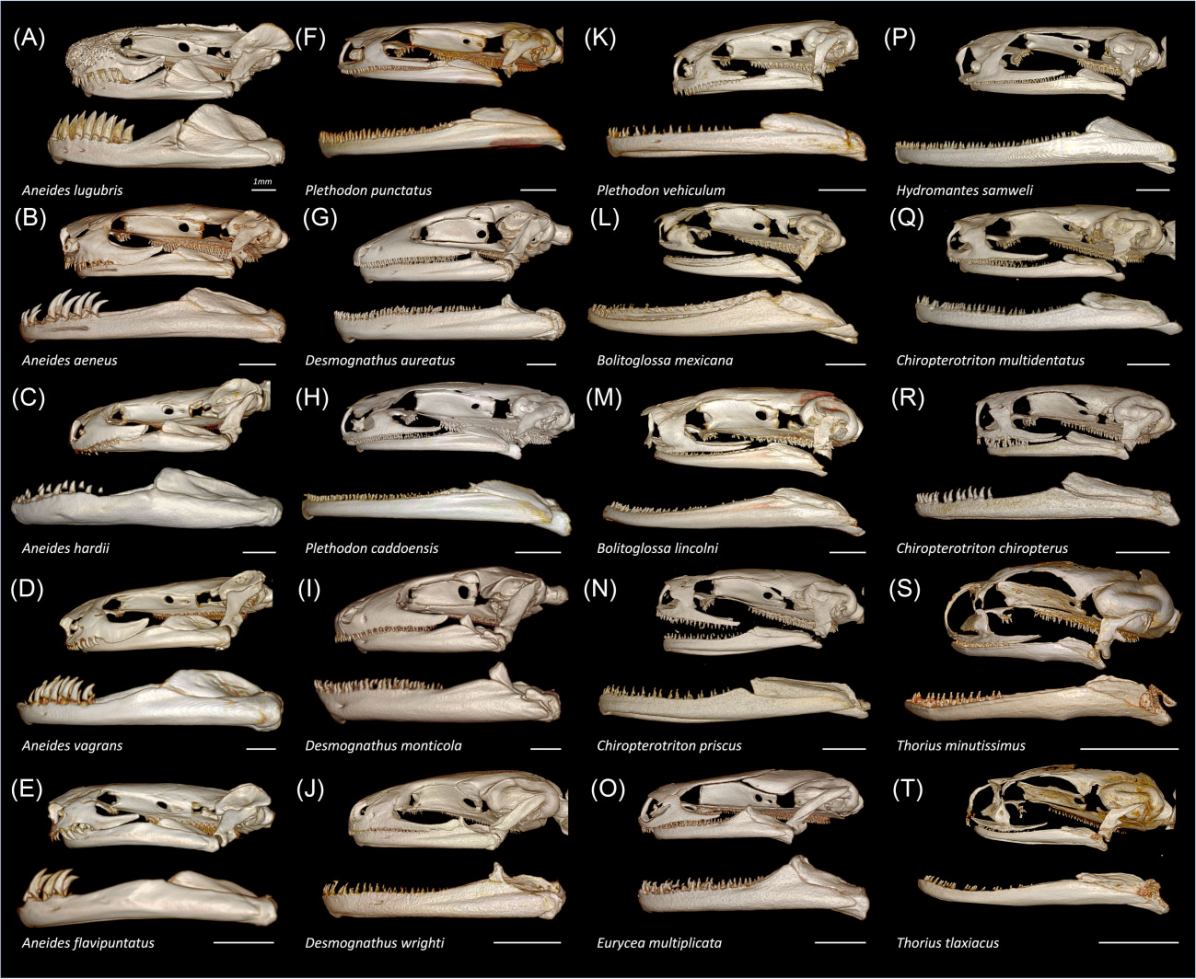
Diversity of plethodontid skulls and teeth across 20 sampled species. Scale set to 1 mm.

**Fig. 5:**
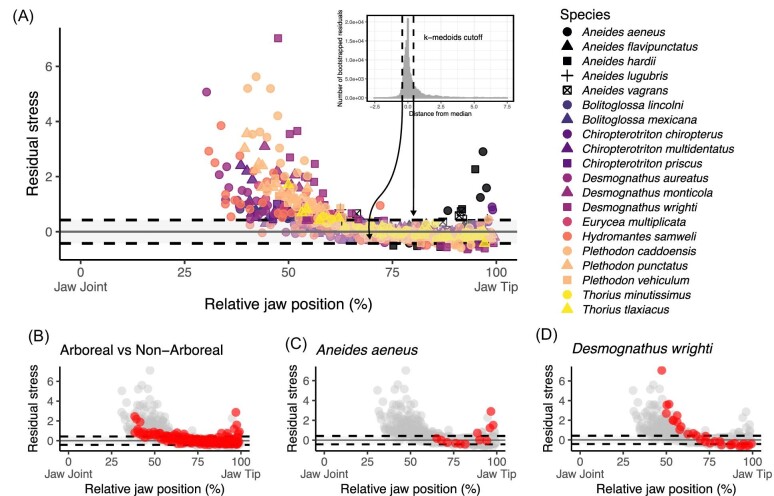
Functional homodonty residual stress values. (A) The residual stress for 780 teeth across 20 salamander species. Teeth outside of the threshold are functionally heterodont, as determined by a bootstrapping analysis depicted by the inset. (B) Residual stress of arboreal salamanders (red) compared to non-arboreal species (gray). (C) Residual stress values of the arboreal *Aneides aeneus* (red), with an anterior patch of functionally heterodont teeth. (D) Residual stress values of *Desmognathus wrighti* (red), depicting its functionally heterodont teeth with high stress values in the posterior tooth bearing region of the jaw.

Plethodontid teeth are mostly conical and vary little in shape or size within an individual and between species. This is true except for *Aneides* salamanders whose teeth range from small cones to large, recurved fangs to flat blade-like teeth (Fig. 4). The number of teeth and their distribution along the jaw vary substantially across the sampled plethodontid salamanders (Supplementary Table S1). For example, *Aneides flavipunctatus* (Fig. 4) has only four teeth on its lower left jaw and they are all located at the anterior margin. Meanwhile, *Desmognathus aureatus* (Fig. 4) has 42 teeth on its lower left jaw that are distributed evenly across the full length of the dentary. *Plethodon caddoensis* (Fig. 4) also has 44 teeth on its right jaw across the dentary but are more densely packed anteriorly. In general, we observed that teeth located posteriorly on the jaw had higher stress values than those located anteriorly (Fig. 5A, Supplementary Table S1). This is an expected result given that the muscle out-lever is shorter and mechanical advantage is higher in the posterior region of the jaw (Westneat 2003).

**Fig. 6:**
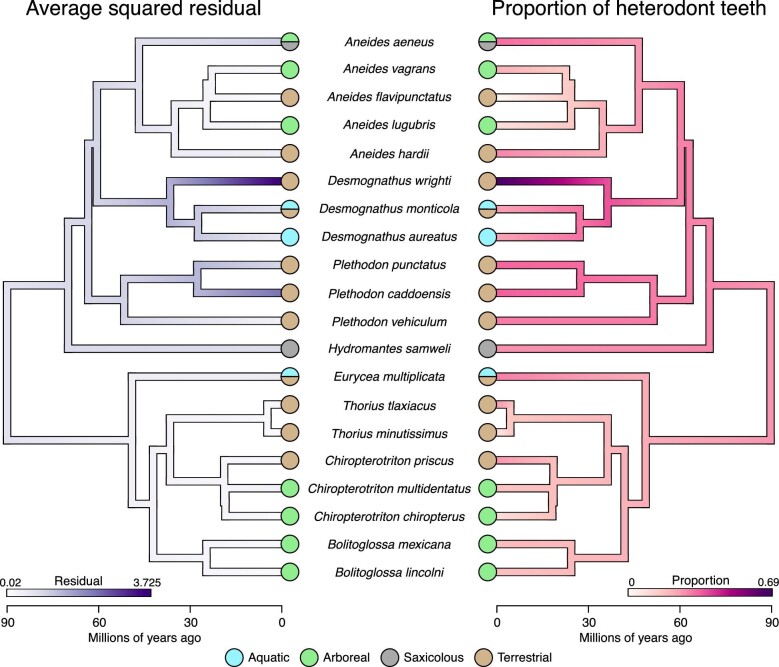
Phylogeny comparing the lower jaws of 20 plethodontid salamanders in different habitats. The left phylogeny depicts the average squared residual that represents the amount of tooth stress variation within a dentition. The right phylogeny depicts the proportion of functionally heterodont teeth on the lower jaw.

Of the 20 plethodontid salamanders sampled, all but one individual (*A. flavipunctatus*), have at least one functionally heterodont tooth (Fig. 5, Fig. 6, Supplementary Table S1). We observed two strategies for evolving a functionally heterodont dentition among our samples. The arboreal *A. aeneus* achieves functional heterodonty through a relatively high proportion of functionally heterodont teeth (∼30%) but a low average squared residual (Figs. 5C and 6). The teeth of *A. aeneus* show strong functional regionalization with an anterior patch of small functionally heterodont teeth and a more posterior patch of large, functional homodonts (Figs. 5C and 6). Initially, these teeth appear to play a role in capturing large and elusive prey. However, *A. aeneus* primarily feeds on small insects (i.e., beetles, mosquitos, and ants) (Lee and Norden 1973) and uses its protrusible tongue to bring prey directly into its mouth, relying little on their large canines (Deban et al. 2020; pers. obs.). When not in trees, *A. aeneus* occupy rock crevices and both males and females will snap at and act aggressively towards intruders (Cupp 1980; Waldron and Humphries 2005). Thus, the tooth morphology and function of *A. aeneus* may stray from the ideal shapes for feeding, and instead reflect the selective pressures of intraspecific combat and territorial disputes. The role of prey retention and processing may be shunted to the vomerine and palatine teeth.

In contrast, the terrestrial pygmy salamander, *Desmognathus wrighti*, has a dentition with a high average squared residual and a high proportion of functionally heterodont teeth (Figs. 5D and 6). Some of its teeth have stresses up to 8x the average residual stress, which are all located in the back of the jaw, while its other heterodont teeth are below the threshold and in the front of the jaw. Where the oral dentition of *A. aeneus* may have a minimal role in foraging, the oral teeth of *D. wrighti* are likely involved in prey capture and processing. Similar pygmy *Desmognathus* species, and presumably *D. wrighti*, feed on small arthropods (mites and springtails) that are shielded in a hard chitin exoskeleton (Bruce 2019). The presence of exceptionally high-stress, posteriorly placed teeth in the dentition of *D. wrighti* could indicate an adaptation towards durophagy. Biting, chewing, or eating hard prey requires teeth able to handle large and variable stresses. Additionally, *Desmognathus* salamanders have a suite of muscular modifications that greatly increase their maximum bite forces compared with most other plethodontids (Deban and Richardson 2017).

We show that even within primarily conical dentitions, there are several strategies of achieving functional heterodonty, each with its own advantage (Fig. 6). Our data do not support the hypothesis that the morphological changes in *Aneides* tooth shapes are accompanied by functional shifts, nor are there similarities among different arboreal lineages (Fig. 5A, B). It may be that the metrics we used to evaluate dentitions do not fully capture the nuances in the function of *Aneides* jaws. However, by examining variation in tooth stress we were still able to detect some outlying dentitions (*A. aeneus* and *D. wrighti*) that have plausible ties to ecology. Incorporating musculature data into the *Dental Dynamics* analysis will continue to provide a clearer picture about the functional capabilities of the jaws and enable more accurate comparisons of bite force and tooth stress. For example, *Aneides* salamanders vary in their degrees of sexual dimorphism (i.e., jaw muscle size and tooth count) and the extent to which they display aggressive behaviors (Wake 1963; Wake et al. 1983, Staub 1993, 2021). Comparing the tooth function of male and female salamanders may help to better explain the evolution of the novel dentitions found in *Aneides* salamanders. Broadly speaking, future work should take a more nuanced approach to study how the evolution of salamander dental morphology is correlated with both feeding and non-feeding behaviors.

## Conclusions

*Dental Dynamics* represents a significant advancement in our ability to quantify and analyze teeth and jaws in 3D. Most vertebrates have teeth, and the vast array of available 3D data sets opens exciting opportunities for reusing CT data in the field of dental morphology. One key advantage of this approach is its ability to quickly quantify morphology, even in the case of small and intricate teeth. *Dental Dynamics* not only simplifies the process of obtaining numerical data but also increases our ability to address large-scale questions related to dental evolution. We demonstrate the power of *Dental Dynamics* to facilitate the functional homodonty method, but its application does not stop there. The output variables of *Dental Dynamics* can be adapted to support the calculation of other metrics or used in tandem with other techniques. For example, methods relating aspect ratio of conical teeth to the energy of puncture may benefit from tools like *Dental Dynamics* that can calculate aspect ratio for many teeth at a time (Zhang and Anderson 2022, 2023). Tools like MandibLever (Westneat 2003) can dynamically model the bite and jaw mechanics more comprehensively than *Dental Dynamics* but do not consider the dentitions. Alternatively, methods such as OPCR or finite element analysis (Evans et al. 2007; Berthaume 2016; Spradley et al. 2017) that focus on characterizing teeth with complex shapes could be used to inform how to make *Dental Dynamics* more useful for modeling the stress of non-conical teeth. That is the beauty of open-source software. *Dental Dynamics* can be continually improved upon to meet the demands of growing needs beyond the scope of its original design. Teeth have been telling the stories of vertebrates for millions of years, our ability to quantify these changes in development, shape, and evolution is paramount.

The utility of *Dental Dynamics* extends beyond CT scans and can be used to analyze 3D surface scans, photogrammetry models, or even pre-segmented models exported from other CT processing software. With the suite of native tools in 3D Slicer, segmenting individual teeth is streamlined and many data modalities may be used with *Dental Dynamics*. The publicization of Slicer extensions and modules such as *SlicerMorph, Dental Dynamics*, and *SegmentGeometry* should inspire other morphologists and biomechanists to derive their own open-source modules or expand upon existing ones. These extensive tools kits will in turn expand the amount of information we can gather from a single CT scan and decrease the amount of time it takes to analyze multiple scans. By packaging the *Dental Dynamics* and *SegmentGeometry* modules together to form the *SlicerBiomech* extension, we have laid the groundwork for an extensive toolkit designed to aid in the collection of biomechanical data from CT scans and other forms of 3D data in the 3D Slicer environment.

## Supplementary Material

obae015_Supplemental_Files
