# Supplementary material for: Dental Dynamics: A Fast New Tool for Quantifying Tooth and Jaw Biomechanics in 3D Slicer
Source: Integr Org Biol. 2024 May 10;6(1):obae015. doi: 10.1093/iob/obae015 (PMC11263487; doi:10.1093/iob/obae015)
Supplement: obae015_Supplemental_Files [file obae015_supplemental_files.zip › Dental Dynamics Tutorial.pdf]

Dental Dynamics Tutorial

The *Dental Dynamics* module is used to model vertebrate jaw function and calculate several key jaw and tooth traits. The module requires a segmentation file containing individually segmented teeth and user-defined anatomical landmarks (jaw joint, tip of the jaw, and the insertion and origin site of up to three jaw closing muscles). These inputs are used to automatically calculate jaw length and muscle in-levers, and for each tooth its position along the jaw, height, width, aspect ratio, surface area, mechanical advantage, output force, and tooth stress. *Dental Dynamics* can also take additional user inputs to estimate muscle parameters such as input force and insertion angle to provide more informed estimates of bite force.

To use *Dental Dynamics*, first go to the *Sample Data* module and download the “Demo Skull”, “Demo Segment”, and the “Demo Jaw Points”. In this example, we will be using data from a CT scan of the arboreal salamander (*Aneides lugubris*), originally obtained from MorphoSource.org.

Then find *Dental Dynamics* module under the SlicerBiomech module menu folder and:

1. Start by entering in the **Specimen Data**. In this case, we will be analyzing the lower left jaw of *Aneides lugubris*.

2. Then select “Dental Dynamics Demo Segment” as the **Tooth Segmentation**. The “Dental Dynamics Demo Segment” may have already been selected by default.

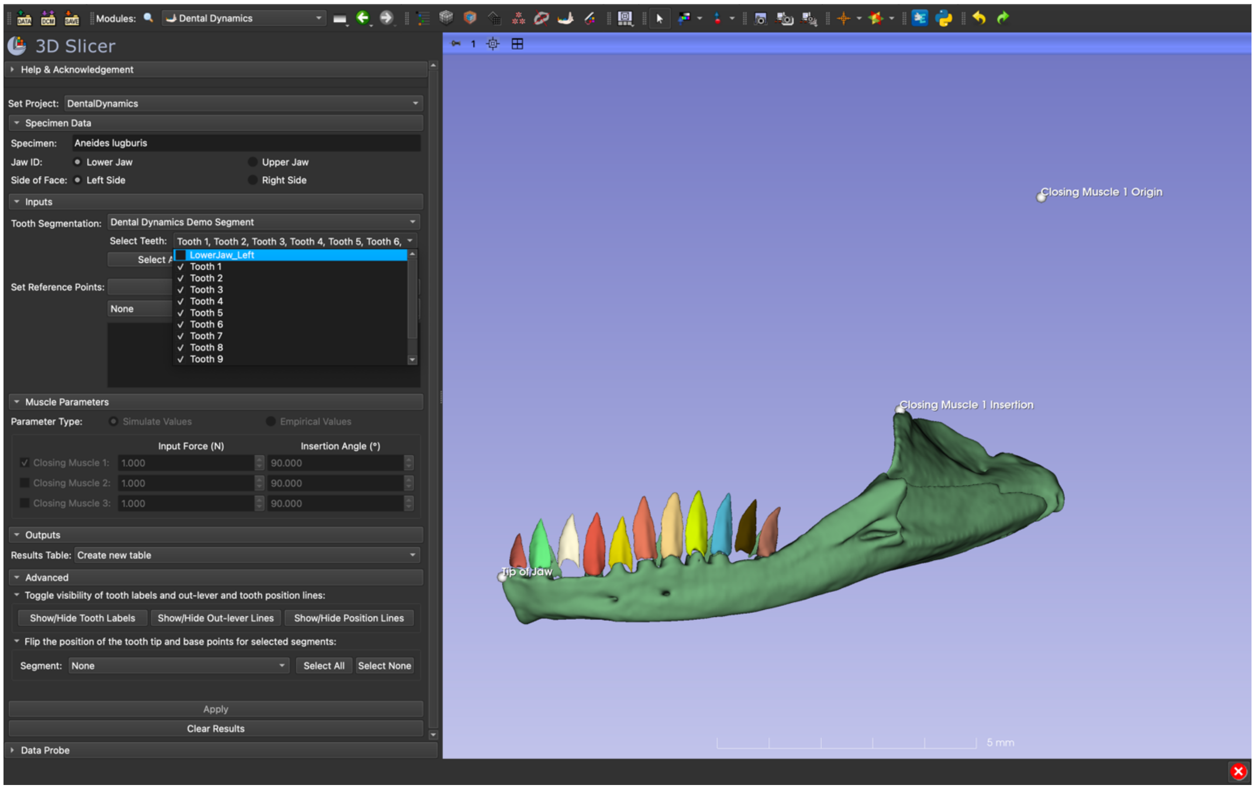

3. **Select Teeth** to perform computations on. Here we will unselect the "LowerJaw_left" segment. You may use the **Select All** or **Select None** buttons to quickly select or deselect segments.

4. Under **Set Reference** Points select the “Dental Dynamics Demo Jaw Points”. If you are using your own data for the first time, click the **Create new reference point list** button to create a blank point list.

5. The *Jaw Joint*, *Tip of Jaw*, *Closing Muscle 1 Insertion*, and *Closing Muscle 1 Origin* points are all predefined in the sample data. Clicking the label name of undefined points will activate point placement mode. To clear unwanted points, enter the position as 0,0,0 or right-click the point in the 3D view and select "Clear control point position".

6. Change the **Input Force** and **Insertion Angle** to simulate bite force with. Retaining the default values (1 and 90, respectively) will ensure tooth force calculations reflect variation in mechanical advantage.

7. If more detailed information about muscles is known and the origin of at least one closing muscle is defined, select **Empirical Values** as the **Parameter Type** to use a different set of inputs. Only values for the selected **Parameter Type** will be used during calculations.

8. The option to add additional closing muscles and change their parameter values will remain disabled (greyed out) until the insertion point (Simulate) or the insertion and origin points (Empirical) have been defined for that muscle.

9. If no **Results Table** has been selected, a new one will be created by default.
10. Click the **Apply** button to perform calculations. Upon completion of the analysis, a results table should have populated the scene and points should have been placed at the tip (red) and base (blue) of each selected tooth.

11. Visually inspect the points to ensure that Dental Dynamics has correctly identified the tip and base of each tooth. Use **Show/Hide Out-lever lines** and **Show/Hide Position lines** to draw lines from the jaw joint to the tip and base of each tooth, respectively. If there is a mistake, see next section on fixing incorrect tooth points.

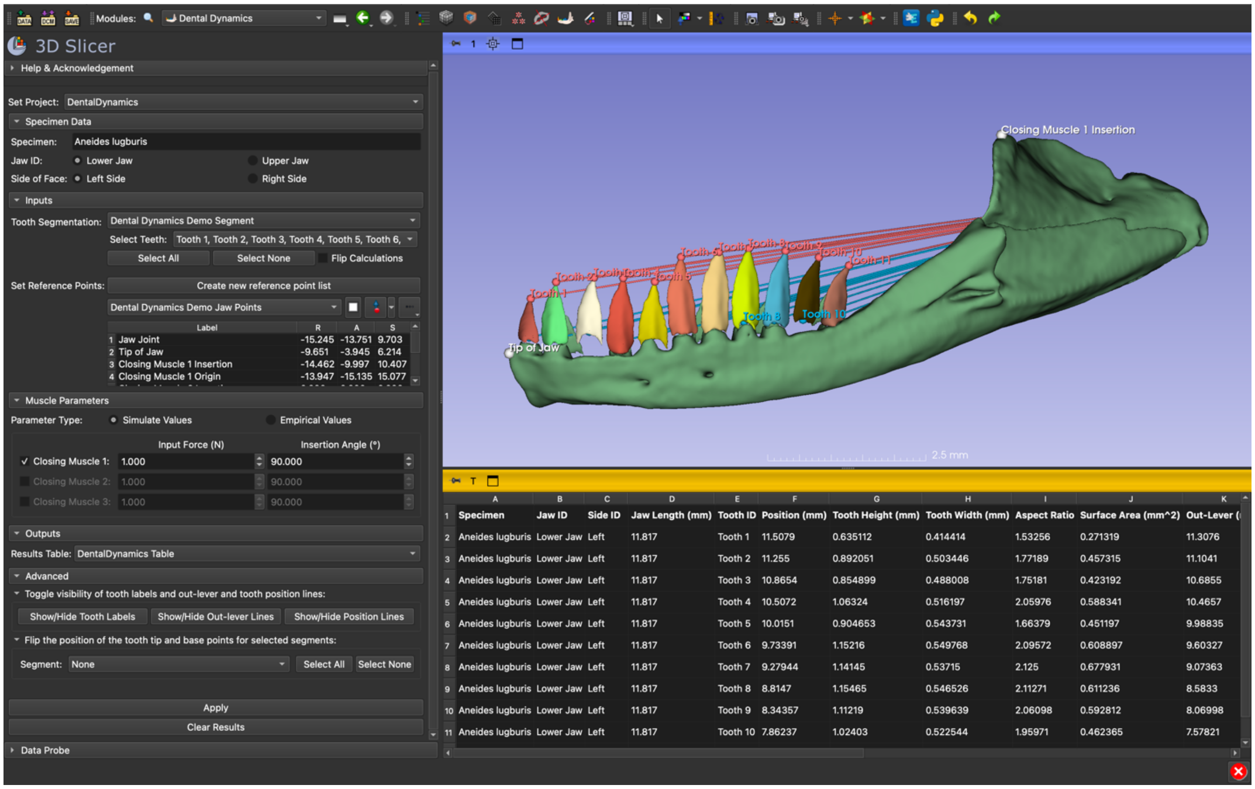

12. Export results by navigating to the *Data* module. Right-click the results table and select "Export to file..." to export as a .csv file. Alternatively, copy cells from the table and paste directly into a spreadsheet.

13. To analyze a new jaw, navigate to **Set Project** at the top of *Dental Dynamics* and Create a new ScriptedModule. Creating new projects for each jaw is recommended when using different reference points and parameters for each jaw.

Fixing incorrect tooth points

Let's say *Dental Dynamics* has incorrectly identified the position of the tooth tip and base for some or all of the teeth on the jaw. If the points are slightly off, they can be manually adjusted and the results table will be updated by clicking the **Apply** button. If the tip and base of some teeth are swapped entirely, use the **Show/Hide Tooth Labels** button to identify which teeth need the tip and base points flipped, and use the **Segment** selector to select which teeth are incorrect and click **Apply** again. If the tip and base points are flipped for all of the teeth, click the **Clear Results** button, select the **Flip Calculations** check box, and click **Apply**.

Using 3D Meshes (.obj, .stl, .ply)

If you would like to use Dental Dynamics with a pre-existing 3D model of a jaw load it into 3D Slicer and navigate to the *Data* module. Right-click your model and select "Convert model to segmentation node". If the model needs additional segmenting, right-click the new segmentation node and select "Export visible segments to binary labelmap" to make a labelmap that can be used as segmentation node's reference volume in the *Segment Editor* module.

Additional Tips

1. If a tooth segment has a hole in it or the base is hollow, then surface area measurements will be overestimated. Use the *SurfaceWrapSoldify* module to make the teeth solid before computing results.

2. Selecting the correct **Side of Face** is somewhat important because it’s used to help discern the tips and bases of the teeth.
